# Higher fibre and lower fat intake is associated with better vascular function in children with type 1 diabetes

**DOI:** 10.1186/1687-9856-2015-S1-O26

**Published:** 2015-04-28

**Authors:** Sarah Toome, Jemma Anderson, Jorien Schokker, Oana Maftei, Roger Gent, Christine Mpundu-Kaambwa, Jennifer Couper, Alexia Peña

**Affiliations:** 1 Women’s and Children’s Hospital, Department of Nutrition, North Adelaide, SA, Australia; 2 Women’s and Children’s Hospital, Endocrine and Diabetes Department, North Adelaide, SA, Australia; 3 The University of Adelaide, Discipline of Paediatrics, North Adelaide, SA, Australia; 4 Women’s and Children’s Hospital, Department of Medical Imaging, North Adelaide, SA, Australia; 5 Women’s and Children’s Hospital, Research and Evaluation Unit, North Adelaide, SA, Australia

## 

Children with type 1 diabetes (T1D) might not consume a healthy diet. A healthy diet is associated with reduced risk of cardiovascular disease (CVD) in adults, but there is no data evaluating the association between diet composition and early markers of CVD in T1D children. We aimed to investigate the macro/micronutrient intakes of T1D children and the relationship with vascular function.

The Australian Child and Adolescent Eating Survey (ACAES–version1.2) Food Frequency questionnaire[1] was administered to 77 T1D children (aged 14±2.3 years, 37 males, BMI z-score 0. ±0.6) participating in an RCT[2], obtaining in-depth macro/micronutrient intake. Vascular function was measured by Flow Mediated Dilatation (FMD) and Glyceryl Trinitrate Mediated Dilatation (GTN). Pearson’s correlation and multivariate regression analysis determined dietary predictors of vascular function.

Children had diabetes duration 5.7±3.9 years, median HbA1c 8.7(range: 6.3-14)% and insulin dose 0.8±0.2 units/kg/day. 37 children used CSII.

T1D children had daily energy intake 10762.3 ±2487.68kJ, protein 113.3±27.68g, fat 88.16±88.16g, carbohydrate 318.60±75.97g, fibre 31.41±8.89g and sodium 3069.91±766.43mg. Better (higher) FMD independently correlated with a higher daily fibre intake (r^2^ =0.25, Coefficient 0.20, p=0.04). Higher daily total fat intake independently correlated with worse (lower) GTN (other GTN associations in Table 1). Daily sodium intake exceeded recommendations of 1500mg, this was not significantly related to FMD/GTN.

**Table 1:** Independent predictors of GTN

R^2^=0.41		
	Coefficient	p-value

Total fats	-0.06	0.02

Vessel diameter	-68.15	0.00

Diastolic BP	0.23	0.04

Pump use	3.35	0.01

T1D Duration	-1.26	0.04

Higher fibre and lower total fat intake, is associated with better vascular function in T1D children. This is the first evidence that diet composition may reduce the risk of CVD in children with T1D in addition to improving diabetes control.
